# Engineered Nanomaterial Coatings for Food Packaging: Design, Manufacturing, Regulatory, and Sustainability Implications

**DOI:** 10.3390/mi15020245

**Published:** 2024-02-06

**Authors:** Oluwafemi Olawore, Motunrayo Ogunmola, Salil Desai

**Affiliations:** 1 Department of Industrial and Systems Engineering, North Carolina Agricultural and Technical State University, Greensboro, NC 27411, USA; oolawore@aggies.ncat.edu (O.O.); maogunmola@aggies.ncat.edu (M.O.); 2 Center of Excellence in Product Design and Advanced Manufacturing, North Carolina Agricultural and Technical State University, Greensboro, NC 27411, USA

**Keywords:** antimicrobial, biodegradable, coatings, humanitarian, nanomaterials, nanotechnology

## Abstract

The food industry is one of the most regulated businesses in the world and follows strict internal and regulated requirements to ensure product reliability and safety. In particular, the industry must ensure that biological, chemical, and physical hazards are controlled from the production and distribution of raw materials to the consumption of the finished product. In the United States, the FDA regulates the efficacy and safety of food ingredients and packaging. Traditional packaging materials such as paper, aluminum, plastic, and biodegradable compostable materials have gradually evolved. Coatings made with nanotechnology promise to radically improve the performance of food packaging materials, as their excellent properties improve the appearance, taste, texture, and shelf life of food. This review article highlights the role of nanomaterials in designing and manufacturing anti-fouling and antimicrobial coatings for the food packaging industry. The use of nanotechnology coatings as protective films and sensors to indicate food quality levels is discussed. In addition, their assessment of regulatory and environmental sustainability is developed. This review provides a comprehensive perspective on nanotechnology coatings that can ensure high-quality nutrition at all stages of the food chain, including food packaging systems for humanitarian purposes.

## 1. Introduction

**Figure 1:**
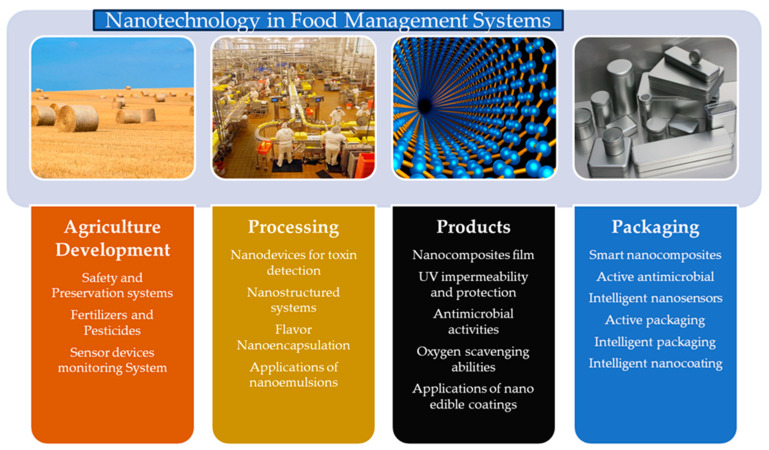
Nanotechnology in food management systems.

A coating layer is defined as a uniform layer that is formed when a material is applied one or more times to a substrate [1]. Typically, surfaces are coated with lacquer or paint to protect them from mechanical, chemical, and weather effects and to improve their aesthetic appearance. Ionomer and coextruded films are used for food packaging lamination and extrusion coatings in all major flexible packaging. In recent years, nanotechnology has become increasingly important in the development of surface coatings. Additives containing nanoscale materials have been used for decades in the production of varnishes and paints (e.g., barium sulfate and iron oxide) [1]. Novel nano-based coatings are widely used for surface functionalization and protection against corrosion and dirt. Designing and developing a coating for a specific substrate, application, and operating environment is a challenge, due to the many influencing parameters such as thickness, grain size, adhesion of the coating to the substrate, hardness, etc. [2,3]. The unique properties of nanomaterials compared to microscale materials have led to the successful development of nanostructured coatings in which at least one component is at the nanometer scale [4,5,6,7]. Nanostructured coatings provide enhanced surface protection and are therefore used in the fields of food packaging and more [8,9]. Different synthetic methods are used to prepare nanocoatings depending on the target application [10,11,12,13,14,15,16]. In addition to the traditional methods of manufacturing nanostructured coatings, such as physical vapor deposition and chemical vapor deposition, new approaches such as laser deposition, the sol-gel method, laser cladding method, spray coating method, and electrodeposition are also used to fabricate nanostructured coatings [17,18,19,20,21,22,23]. With the development of nanomaterials and nanocoatings, corrosion control has become relatively more effective [24]. Nanocoatings are versatile as they are resistant to corrosion, temperature fluctuations, abrasion, adhesion, abrasion, and fogging, and can be biocompatible and antibacterial [9,23,25,26,27,28,29,30,31,32,33,34]. The benefits of nanotechnology in food management systems from food sources, processing, and products to packaging are described in Figure 1.

Food preservation refers to the management and treatment of food to prevent germs from destroying its nutritional content, edibility, or quality. Among the traditional ways that have been used as food preservation procedures are drying, canning, and freezing. To lessen environmental pollution, packing materials are ideally comprised of biodegradable materials. The application of nanotechnology to the food packaging sector has made this concept a reality. When packaging food, several techniques need to be considered, including the use of high-barrier plastics, the introduction of antimicrobials, and contamination detection procedures. Smart packaging uses nanosensors to identify food degradation and release nano-antimicrobials as needed to prolong shelf life. According to Alfadul and Elneshwy (2010), nanoscience can offer solutions for issues like altering the permeation behavior of foils, improving mechanical and heat resistance properties, improving barrier qualities (such as thermal, chemical, and microbial), introducing active antimicrobial and antifungal surfaces, and sensing/signaling any changes that have taken place in the microbiological and biochemical realm. The most widely utilized nanomaterials as antimicrobials in the food business are silver nanoparticles and their nanocomposites [35]. Ag+ ions, which attach to membrane proteins and form pits or induce other morphological changes, are obtained from these nanoparticles [36]. These also trigger the production of reactive oxygen species (ROS) in bacterial cells, which ultimately results in oxidative stress-induced cell death [37]. According to Ntim et al. (2015), there is no discernible or minute amount of silver nanoparticles released from the containers that migrate to the actual food samples and food stimulants, suggesting that silver nanocomposites are quite safe for food packaging [38]. Furthermore, new coatings made of nanomaterials with special qualities are being introduced because of continuous research and development efforts and advances in nanotechnology. Coatings made of nanomaterials frequently minimize environmental impact, increase energy efficiency, and decrease waste, all of which are in line with the current trend toward sustainable practices. In comparison to traditional technologies, the nanomaterial coating market is expected to experience significant growth shortly due to the growing demand for improved coatings and the ongoing development of new applications.

### 1.1. Background of Food Packaging Challenges in Humanitarian Efforts

Global food insecurity remains a pressing challenge, affecting millions of individuals worldwide. Despite advancements in agricultural technologies and food production methods, a significant portion of the global population continues to face inadequate access to safe, nutritious, and sufficient food. According to The United States Department of Agriculture (USDA), food insecurity is defined as “the lack of access to sufficient, safe, and nutritious food [39]”. This definition underscores the multidimensional nature of food insecurity, which encompasses not only the availability of food but also its safety and nutritional quality. The problem of global food insecurity is closely linked to the Sustainable Development Goal (SDG) 2, which aims to “end hunger, achieve food security and improved nutrition, and promote sustainable agriculture 1”. This goal reflects the international community’s commitment to addressing the root causes of food insecurity and hunger while promoting sustainable food systems [40]. The issue of food insecurity is exacerbated by various factors, including poverty, political instability, climate change, and natural disasters. Climate change poses a substantial threat to global food systems. Rising temperatures, unpredictable weather patterns, and extreme events adversely impact crop yields and agricultural productivity. The Intergovernmental Panel on Climate Change warns that climate change-related shifts in precipitation and temperature patterns could lead to a decline in food production by up to 2% per decade. Additionally, a NASA study found that climate change may affect the production of perishable foods as early as 2030, with maize crop yields projected to decline by 24% (Team, by Ellen Gray, NASA’s Earth Science News). The COVID-19 pandemic unveiled new dimensions of food insecurity, with disruptions in supply chains, income losses, and limited access to markets further amplifying the problem. According to Karpman et al., about one-third of US families struggled to meet basic needs during the pandemic [41]. The disruptions in food availability and accessibility led to increased reliance on food banks and nutrition aid programs [42]. It therefore became important for food banks and other hunger relief organizations to bridge the gap in food accessibility by bringing food closer to the communities that need it through various hunger relief programs [43]. Addressing global food insecurity necessitates multifaceted approaches that consider both immediate relief efforts and long-term sustainable solutions [44]. The establishment of meal programs, food pantries, and mobile food markets serve as immediate solutions to a long-standing problem. The food distributed includes fresh food and shelf-stable foods. Nutritious food packages must include fresh produce including fruits, vegetables, dairy, and all kinds of meat. To bring the food closer to underserved communities, the food may need to travel longer distances to rural areas to reach them. It is therefore important to consider the viability of the food to prevent spoilage before it reaches its destination. This is where an effective and efficient food packaging system must be developed and employed to ensure that the food provided for humanitarian purposes is healthy and fresh. Nanoengineering, the branch of engineering that manipulates structures and devices at the nanoscale, has shown immense potential in revolutionizing food packaging and preservation methods. In recent years, the intersection of nanotechnology and food engineering has opened new avenues for addressing these issues. This paper delves into the transformative role of nanomaterials in food packaging, focusing on their applications in the context of food rescue and humanitarian relief. In humanitarian efforts, ensuring the safe transportation and preservation of food is paramount, yet it poses significant challenges [45]. Humanitarian situations often involve transporting perishable goods over long distances and varied environmental conditions, making conventional packaging methods inadequate. Traditional packaging materials lack the necessary barrier properties, leading to quick spoilage and contributing to food waste in emergency aid scenarios. Furthermore, the lack of reliable refrigeration options in disaster-stricken regions exacerbates the problem, demanding innovative solutions to extend the shelf life of perishable foods without compromising safety [46]. Humanitarian organizations are continually striving to enhance food packaging methods to address these challenges. Research in this domain emphasizes the urgency of developing packaging solutions that can withstand harsh conditions and protect food items from contamination, spoilage, and physical damage during transit. The integration of nanoengineered materials in food packaging has emerged as a promising avenue. By leveraging the unique properties of nanomaterials, such as enhanced barrier capabilities and antimicrobial features, researchers aim to create packaging that ensures the safe delivery of food aid, particularly in regions where resources are limited, and infrastructure is compromised. These advancements hold the potential to revolutionize humanitarian food packaging, minimizing waste and maximizing the impact of aid efforts in vulnerable communities.

### 1.2. Antimicrobial Nanomaterials: Safeguarding Food Safety

Foodborne illnesses resulting from microbial contamination pose a significant threat, especially in disaster-stricken areas where resources are limited. Nanomaterials with inherent antimicrobial properties, such as silver nanoparticles and nanoliposomes, have been integrated into food packaging systems to inhibit the growth of pathogenic microorganisms [47]. These antimicrobial nanocoatings create a hostile environment for bacteria and fungi, ensuring the safety of perishable foods during storage and transportation.

### 1.3. Sustainability and Environmental Impact

In addition to enhancing food safety and shelf life, nanomaterials contribute to sustainability efforts by reducing food waste and minimizing the environmental footprint of packaging materials. Biodegradable nanocomposites made from natural polymers and nanoclays have emerged as eco-friendly alternatives to conventional plastic. These materials not only decompose rapidly, reducing landfill burden, but also conserve resources by extending the usability of packaged foods [48]. The integration of sustainable nanomaterials aligns with the principles of humanitarian relief, emphasizing the importance of environmentally responsible solutions. Nanomaterials have ushered in a new era in food packaging technology, offering innovative solutions to the challenges faced by food rescue and humanitarian relief initiatives. By leveraging the unique properties of nanomaterials, the food industry can develop packaging systems that enhance food safety, prolong shelf life, and promote sustainability [49]. As ongoing research continues to explore novel applications of nanotechnology in food packaging, the potential for improving the efficiency and effectiveness of food rescue efforts remains promising.

This paper explores the design and manufacture of nanomaterial coatings for food packaging, including regulatory and sustainability issues. Section 2 compares nanotechnology with traditional packaging methods, evaluates coating technology, and examines current food packaging methods and preservation techniques. Section 3 highlights nanomanufacturing, explores nanomaterials in food packaging, and assesses their impact on food quality and safety. Section 4 focuses on food preservation and the enhancement of shelf life. Section 5 addresses environmental, safety, and regulatory impacts with a focus on sustainable development and regulations. Section 6 deals with humanitarian aspects and explores applications in extreme environments. Finally, the main conclusions and recommendations are presented at the end of Section 7.

## 2. Development and Design

### 2.1. Comparative Analysis: Nanoengineered vs. Traditional Packaging Methods

In comparison to traditional packaging methods, nanoengineered packaging has several advantages, including improved mechanical barrier, heat-resistant properties, and biodegradability [50]. Nanomaterials can be utilized to detect food deterioration using nanosensors due to their increased antibacterial properties [51]. Antimicrobial packaging (structured polymeric films) or encapsulating materials limit the development phase of microorganisms on the packed food’s surface by distributing active substances onto the food or into the external area [52]. Antimicrobial nanoparticles are used in active packaging to protect food against detrimental and spoilage-causing bacteria to extend shelf life and quality freshness. They are also included in the active packaging to make it stronger, lighter, and less O_2_ accessible [53,54,55].

Today’s market is seeing an increase in the use of different materials to meet the growing demand for cost-effective products. There is documented evidence of the adoption of nanotechnology to enhance existing technology and develop new products with improved functions, features, design, characteristics, reliability, and quality. The application of nanotechnology has made existing and new materials become lighter, stronger, and more durable thanks to their mechanical, electrical, and conductive properties. The development of nanotechnology also means many tangible benefits or dangers. The food industry is experiencing a paradigm shift from the traditional way of preserving food to the more efficient nature of nanomaterials designed at the nanoscale level with excellent barriers against oxygen, ultraviolet light (UV), water vapor, gas ingress, moisture, and contaminants. The most interesting question facing developers and researchers around the world is the future of nanotechnology, its application, associated risks, and possible consequences. Every developer and researcher must understand the functional, geometric, and mechanical properties of materials to advance the design process and its application. Before selecting a material for commercial use, the interaction of material, function, form, and environment must be evaluated. Researchers have studied and used nanotechnology in medicine [56,57] to promote, protect, restore, and improve health, and in the energy sector [58,59], to reduce greenhouse gases and improve sustainability. In the electronics industry [60,61] there is a need for faster, smaller, and more efficient handles, while in the food industry, to improve taste, flavor, texture, color, shelf life, and packaging [62].

### 2.2. Active Packaging System

**Figure 2:**
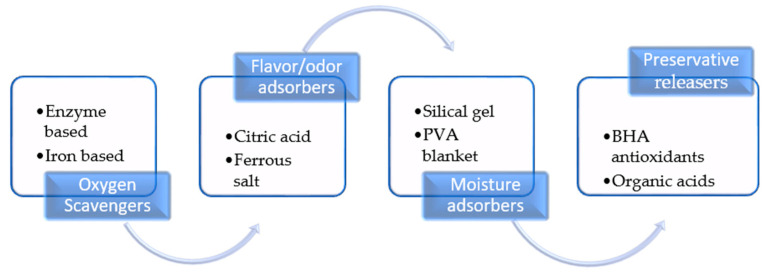
Active packaging system.

When specific additives are integrated into packaging film to extend the shelf life of food products, the package composition is considered active [63]. This active food packaging can seek out moisture, oxygen, odor taints, antioxidants, preservatives, antimicrobials, enzymes, etc. [64]. The creation of active packaged food materials involves adding active compounds to the template of existing packaging materials or immobilizing antioxidants on the surfaces of the packaging film to improve its functionality [65]. Figure 2 describes several foods that have benefited from active packaging technology.

### 2.3. Overview of Coating Technology

Designing a nanotechnology platform involves integrating, formulating, and using cost-effective methods such as roll-to-roll nanocoating, thin-film spray coating, extrusion-based coating, or alloying [66]. The purpose of coating materials is to protect, reinforce, and/or provide additional functions and properties to the underlying object’s surface or bulk materials. In recent years, nanotechnology has been widely applied to functional coatings, because nanoscale processing materials can provide unique chemical, mechanical, thermal, surface, and photophysical properties that can be exploited to improve functions required in urban environments [67]. Research shows that active ingredients can be incorporated into active packaging coatings using immersion, non-covalent immobilization, and layer-by-layer coating methods and techniques. The surface properties of materials, especially packaging materials, are different from bulk materials [65]. Photocoating adds several properties to the surfaces of materials that enable the materials to be used in functional packaging systems, including antimicrobial and self-cleaning [68], antistatic and self-protective [69], metal chelating antioxidant [70], free-radical removal antioxidant [71], biocatalytic [72], and easy printing properties [73]. Functional coating materials vary in their properties and functionality from organic polymers to hybrid composites and inorganic nanoparticles. The research and development of functional coating technologies mainly involves the development of coating materials and coating methods based on different applications [74]. The study of functional coating technologies has attracted increasing interest in recent years due to their promising application in advanced engineering materials. The traditional approach involves applying a coating to the surface for environmental protection or aesthetic properties. Recently, the growing demand for technical universal materials has encouraged the development of innovative, intelligent, and high-performance coatings in various fields of application. These materials must be designed for use in different industrial contexts or for specific applications, offering targeted properties such as resistance to harsh environmental conditions, chemothermal and mechanical stability, tailored surface morphology, or environmental sustainability [75]. The main goal of nanotechnology platform development is to significantly improve the barrier properties of biomaterials, such as packaging papers. The packaging material must have properties that prevent the migration of various substances that penetrate or permeate the atmosphere [76]. A polyvinyl alcohol (PVA)-based polymer coating was used to improve the barrier properties of the paper through its good film-forming ability [77]. The coated paper had excellent water resistance due to a hydrophobic contact angle of about 100°. After PVA/AKD coating, the grease resistance and mechanical properties of the base paper also improved [78]. Sensors have been developed that detect changes in oxygen, mechanical tearing, temperature, and pH to ensure the multi-functionality of food packaging. Nanoparticles are used in the development of advanced packaging, active packaging, and smart packaging that help preserve food and traceability throughout the supply chain. Nanoparticles have antimicrobial activity, oxygen scavenging ability, UV transmission, and many other properties that make them valuable for use in nanocomposites [79].

### 2.4. Plastic Film Coating

Coating as a method to improve the properties of plastic films and containers is an active area of innovation. Coatings are applied to the surfaces of plastic films to improve heat sealing and sealing properties. For example, acrylic jars are available in gold, gray, and clear and are suitable for a variety of products including soups, vegetables, broths, and nutritional drinks. Acrylic coatings have good odor-barrier properties. The coating is glass-clear, hard, heat-sealable, and very glossy. It is highly resistant to oxygen, aroma, and gas [80,81]. Due to environmental concerns that organic products like PVOH coatings may produce dioxins, the compound ignites, and has a high gas barrier, nanotechnology is being employed to replace polyvinylidene dichloride (PVDC) without changing the coating parameters [80]. Liquid-phase treatment of packaging coatings with nanoclay and polyvinyl alcohol (PVOH) has been developed [82]. The resin-based ionomer coating (LTSCs) is used in emulsion form in contrast to acrylic and PVDC coatings. The ionomer surface inks well without leaving any printing ink residue. SiOx is added as a coating. SiOx is transparent, reusable, and recyclable and has good protective properties. This is a technology that deposits silicon oxide (SiOx) on plastic films [83]. The photolysis of SiOx (glass) is like that of aluminum oxide during vacuum casting, as the resulting material is relatively simple. As with aluminum, the addition of a SiOx layer significantly improves the air and barrier properties and has the benefit of making the coating transparent [83,84,85].

### 2.5. Research Area of Nanomaterials Applied in Coatings

Researchers around the world have studied the behavior of functional coatings using nanoparticles. This includes scratch resistance, pollution resistance, self-cleaning, corrosion protection, and UV protection. For example, Hasim et al. (2015) looked at UV/ozone-treated commercial low-density polyethylene (LDPE) films coated with layer-by-layer (LbL) alternate deposition of polyethyleneimine (PEI) and poly (acrylic acid) (PAA) polymer solutions and antimicrobial silver [86]. They showed that the resulting films containing antimicrobial Ag NPs can be studied in antimicrobial packaging. An et al. (2008) documented that coating fresh asparagus spears with AgNP/polyvinylpyrrolidone nanocomposite films extended their refrigerated shelf life by 25 days [87]. In addition, cellulose cushions containing silver nanoparticles have also been successfully used to cover beef, with a significant reduction in microbial load [88]. Chawengkijwanich and Hayata (2008) observed a 3-log reduction in the incidence of Escherichia coli *E. coli* after 3 h of illumination compared to fresh lettuce treated with an oriented polypropylene (OPP) film coated with TiO_2_ nanoparticles [89]. In contrast, uncoated films can reduce the number of *E. coli* by only 1 log under similar conditions. Another study analyzed plastic films coated with TiO_2_ nanoparticles for *Penicillium expansum* spoilage of apples, tomatoes, and lemons. The results showed that the growth of *P. expansum* was inhibited due to the photocatalytic properties of TiO_2_ particles under the influence of light [90]. Chen et al. (2013) modified the paper to form a lotus-like superhydrophobic surface by coating it with R812S silica nanoparticles and polydimethylsiloxane (PDMS) silicone oil [91]. The coated paper had strong waterproof properties. Maneerat and Hayata (2006) developed TiO_2_-coated polypropylene films to remove ethylene vapors from packaged horticultural products. Smolander et al. (2004) detect spoilage of meat products by applying a transition metal coating (silver or copper) (1–10 nm thick) to plastic film or paper packaging structures [92]. When using graphene materials in food packaging, GM has been incorporated into foods or coatings to provide antimicrobial activity and has been used as fillers to improve the physical properties of the films or coatings [93]. Food packaging based on nanotechnology offers many advantages over conventional food packaging materials, improving several properties such as temperature resistance, better durability, flame resistance, sealing, recyclability, and optical properties, as well as processability due to lower viscosity. The expert delivery of active materials into biological systems at low cost reduces environmental damage. Such advances make it an ideal candidate for the development of nanomaterials in a wide range of food packaging applications, such as processed meat and meat products, cheese, confectionery, cereals, and convenience foods. It also helps in extrusion coating applications for fruit juices and dairy products or co-extrusion processes for bottling beer and carbonated beverages [94,95].

Due to the importance of food packaging, many authors have recently investigated different types of nanoparticles [96,97,98]. Their work showed that the interaction of silver nanoparticles (AgNPs) with DNA helps to detect and treat the degradation process, considering the prevention of microbial attack and the possible functional nutrition of coconut water [96]. Several studies have shown that nanoparticles such as gold and silver extend the life of food packaging due to their ability to prevent and reduce microbial contamination [96,97,98,99,100]. Toker et al., 2013 reported that Zn, Ti, Cu, Au, and Ag are emerging metal nanoparticles with biocidal properties for use in food packaging [101].

### 2.6. Manufacturing of Coatings

Nanocoatings require a narrow particle size distribution. The so-called sol-gel technique plays an important role in the production of nano-coatings. In this process, the sol-gel is applied to the surface using a conventional coating method (e.g., dip, spray, or spin coating). The thickness of the resulting layer is 0.5 to 3 μm. The use of nano-coatings on external surfaces is based on the use of organic nanocomposites as binders in water-based surface coatings. The nanocomposites are prepared by emulsion polymerization of acrylates in silica sol. When utilizing this method, a uniform distribution of silica nanoparticles in the polymer and silica content of up to 50% in the nanocomposite is possible. Organic components greatly improve the strength and durability of the coating. In addition, the surface acquires superhydrophobic properties due to the high concentration of polar silanol groups. These properties create a self-cleaning effect on the surface [55].

## 3. Nanomaterials in Food Packaging

### 3.1. Current State of Nanoengineering Applications in Food Packaging

Nanocomposites used in food packaging applications are classified into three main functions (i.e., advanced, intelligent, and active food packaging) [79]. Advanced packaging involves the use of nanoparticles in nanocomposite materials to improve their mechanical properties, barrier properties, and stability under different temperature and humidity conditions [55]. Smart packaging technology uses interactive technology to provide and improve real-time information about the quality of packaged foods and marketing strategies. In addition, it protects against fraud and counterfeit products and indicates exposure to some harmful factors such as insufficient temperature or high oxygen levels [102,103]. Active packaging provides protection and preservation based on mechanisms activated by natural or acquired factors (antimicrobial activity, biodegradable activity) and reduces food loss due to their shelf life extension [104]. Although many studies on new applications of nanomaterials in food packaging are reported every day, most of the materials are still at the stage of feasibility and demonstration studies, and employment in the food packaging industry has not yet been approved due to safety issues, which may be due to the transfer of nanomaterials from the packaging to the food matrix [105]. In general, nanomaterials used for food packaging can be divided into two categories: inorganic and organic materials. In previous materials, metals, metal oxides, and clay nanoparticles embedded in nanocomposite films and nanofibers [53,106] were discussed. Furthermore, some inorganic materials such as oxidized nanoparticles (CuO, ZnO, TiO_2_, MgO, and Fe_3_O_4_) have attracted much attention. The interest in these oxides stems from their ability to withstand harsh processing conditions and enhance strong inhibition against foodborne pathogens. Clay can resist gases, water vapors, and improve the mechanical strength of biopolymers [107]. The second group consists of organic materials including, but not limited to, phenols, halogenated compounds, quaternary ammonium salts, plastic polymers, and natural polysaccharide or protein materials such as chitosan, chitin, zein, and whey protein isolates, which have recently been very highly rated [108,109]. The food industry has begun to use nanotechnology to develop nano-sized ingredients to improve the color, texture, and taste of foods [62,110]. TiO_2_ and SiO_2_ nanoparticles [111,112] and amorphous silicon dioxide [112,113] are used as food additives. TiO_2_ is used as a coloring agent when coating donuts with icing sugar. The main nanomaterials used for food packaging are montmorillonite (MMT), zinc oxide (ZnO-NP) coated silicate, kaolinite, silver NP (Ag-NP), and titanium dioxide (TiO_2_NP), because these nanomaterial-coated films form a barrier to oxygen, carbon dioxide and aromatic compounds [114]. In addition to the well-known silver nanoparticles and nanoclay, the nanomaterials used in the package include nanometal oxides [115], nitrocellulose [116], and halloysite nanotubes and essential oils [117]. These nanomaterials can provide various functions to packaging compounds, including antioxidant (e.g., essential oils), antimicrobial (e.g., nano-silver), ethylene (e.g., nano-KMnO_4_), and oxygen scavenger (e.g., Pd- nanoparticles) functions, as described in a recent review article [118]. The selection of the main nanomaterials used in food contact materials is described in Table 1.

Studies show that nanocoatings, a good substitute for traditional polystyrene, polypropylene, and other coatings used in food packaging and storage, prevent the formation of bacteria. Additionally, less waste is produced by microbes. The most often utilized nanocoating materials for food preservation and packaging are titanium dioxide and silicon dioxide [127]. For example, silicon dioxide is used as a food colorant, anticaking agent, and drying agent. Further, nanostructured titanium dioxide can be utilized to eradicate diseases and germs on surfaces that come into contact with food due to its potent photocatalytic activity. One of the most significant characteristics of nanomaterials is their surface-to-volume ratio, which is also responsible for many other characteristics. The unique physio-chemical properties of these high surface-to-volume ratio nanomaterials, including their solubility, toxicity, strength, magnetism, diffusivity, optics, color, and thermodynamics, are displayed by these high surface-to-volume ratio nanomaterials [128,129]. Materials at the nanoscale can interact with a greater number of biological molecules and microbes, more successfully exhibiting their antibacterial capabilities due to this high surface-to-volume ratio.

### 3.2. Relevance of Nanomaterials for Food Packaging

Nanomaterials have garnered significant attention in the food packaging industry due to their potential to address emerging challenges and improve sustainability. This literature review provides an overview of the contributions of various academic research papers to the field of nanomaterials for food packaging and sustainability.

In the editorial “Nanomaterials in Food Packaging”, Garcia discusses the development of composites using nanomaterials and their impact on food packaging. It references several relevant studies on the applications and implications of nanotechnology in the food sector, providing insights into the potential of nanomaterials for creating advanced food packaging with enhanced barrier properties and active packaging capabilities [130]. Alweera et al., highlight the benefits and drawbacks of using nanotechnology in food packaging, emphasizing its ability to enhance the properties of food packages and extend the shelf life of food products [79]. Sharma et al., emphasize the evolving nature of food packaging considering consumer demands for natural quality, safety, minimal processing, extended shelf life, and ready-to-eat concepts. They also discuss the technological advances in food packaging, particularly in the domain of nanomaterials, shedding light on the future applications of nanotechnology in food packaging [55].

The paper “Nano-Food Packaging: An Overview of Market, Migration Research” reviews the availability of nano-food packaging in the current market and reports on case studies of nanomaterial migration. In this paper, Bumbudsanpharoke and Ko provide an in-depth exploration of the technological advances in food packaging, particularly in the domain of nanomaterials, and their implications for sustainability and food quality. They also examine different regions of the world and the status of the regulations there. While the United States stands at the forefront of pioneering nanomaterial safety measures for food and its packaging, Canada lacks specific regulations on nanomaterials. Health Canada, however, leverages existing legislative and regulatory frameworks to advance public health goals and mitigate potential health risks associated with nanomaterials [131].

In their study, Ahmad et al., delve into the potential of nanotechnology as a promising and widely used resource in the food packaging industry, shedding light on the types of nanomaterials being used and their societal concerns. Their study suggests that the remarkable physicochemical properties, bioavailability, and chemical reactivity inherent in nanomaterials may potentially lead to a significant level of cytotoxicity [132].

### 3.3. Effect of Nanomaterial Composition on the Improvement of Barrier Properties

Nanoparticles can be classified based on their size, shape, and physical and chemical properties. The classification of these nanomaterials often determines their functionality. Due to their unique structure, nanomaterials offer many advantages in improving barrier properties, such as impermeability, thickness, nanoscale sensors, etc.

#### 3.3.1. Impermeability

Conventionally, packaging materials have been limited in their ability to provide comprehensive protection against environmental factors that can compromise product quality and safety. The ability to manipulate the structure and composition of materials at various nanoscales promises to create effective barriers for food packaging applications. Nanoscale structures are designed to be very dense and impermeable. For example, to maintain the composition of the gas in the package, the structure of the package material must be impermeable to gases [108].

#### 3.3.2. Thickness

The thickness used in certain packaging applications varies and may have commercial implications. Nanomaterial protective shields offer the same protection as thicker traditional materials, including reduced thickness with less material usage [133]. Lower thickness coatings can be vital for transportation in humanitarian food logistics wherein bulkier materials can limit the distribution of perishable items under climate-controlled environments.

#### 3.3.3. Nanoscale Sensors

Nanotechnology is used to maintain and extend the sensory quality shelf life of food. Throughout the life cycle of food, nanoscale sensors are added to packaging materials to help detect and measure properties such as the temperature, humidity, gas content, and freshness of perishable products. They are vital in identifying contamination from microbes to check the condition of food during transportation and storage. Real-time data from these sensors has helped suppliers, manufacturers, retailers, and consumers make timely quantitative decisions [134].

### 3.4. Chemically Released Nanopack

The possible release of nanomaterials from coatings has been the subject of several studies [135,136,137,138]. Particles smaller than 100 nm are released during the abrasion, sanding, and aging processes. However, the added nanoparticles are usually retained in the binder matrix. Packaging can release nanoscale antimicrobials, antioxidants, flavors, aromas, or nutrients into foods or beverages to extend shelf life or improve flavor or aroma [139,140,141,142]. Many experts agree that the release of detached nanomaterials from the coating can only be carried out by the prechemical or thermal treatment of the matrix material, not by mechanical treatment [128]. Studies have shown that small synthetic TiO_2_ particles with a size of 20–300 nm or silver nanoparticles with a size of less than 15 nm can be released from exterior paint during the weathering process [143,144].

### 3.5. Nanostructured Materials in Food Packaging

Functional properties of bionanocomposite films such as starch, cellulose, chitosan, montmorillonite (MMT), metals, and metal oxides have been used in food packaging. Nanomaterials as reinforcements can improve the mechanical, mechanical, and thermal properties of composites, creating new and better materials. In addition, nanomaterials can be used to develop active ingredients with antibacterial, antioxidant, and other effects [145]. In addition to the well-known silver nanoparticles and nanoclay, the nanomaterials used in packaging are nanometal oxides [116], nanocellulose [117], halloysite nanotubes, and essential oils [118]. These nanomaterials can provide a variety of functions to cap compounds, including antioxidant (e.g., essential oil), antibacterial (e.g., nanosilver), ethylene scavenging (e.g., nano-KMnO_4_), and oxygen scavenging (e.g., Pd nanoparticles) according to recent review articles [146].

Although the exact mechanisms underlying the antibacterial properties of nanoparticles remain unclear and depend on the microorganism type, oxidative stress induction, metal ion release, and non-oxidative mechanisms are currently recognized as viable options. Against both Gram-positive and Gram-negative bacteria, nanoparticles have shown broad-spectrum antibacterial properties. ZnO nanoparticles, for instance, have been shown to inhibit *Staphylococcus aureus* [147]. Based on current research, the following primary mechanisms underlie nanoparticles’ antibacterial effects: the bacterial cell membrane can be disrupted, reactive oxygen species (ROS) can be produced, the bacterial cell membrane can be penetrated, and intracellular antibacterial effects, including interactions with DNA and proteins, can be induced.

It has been effectively shown that the size, dose, concentration, and shape of nanoparticles determine their actions. Generally speaking, antiviral substances work against viruses directly or by obstructing critical stages in their replication [148]. For example, silver nanoparticles have been shown to have potent antibacterial properties as well as to be effective against a variety of viruses. Despite the fact that the exact mechanism of antiviral action is not completely clear, silver nanoparticles can directly affect viruses and the initial stages of their interaction with the host cell. This is dependent on a number of factors, including functionality, size, shape, and concentration. Additionally, AgNPs’ broad-spectrum antiviral activity and capacity to stop cell infection have drawn a lot of interest in the food packaging industry. AgNPs primarily function by physically interacting with the free viral particle, as numerous studies have shown. AgNPs have the ability to impede the initial stages of viral replication or to have virucidal effects [149]. However, it should be highlighted that the studies used to elucidate the mechanisms of action of AgNPs are heterogeneous, which can occasionally make it challenging to identify the stage of viral replication that is inhibited [150].

#### 3.5.1. Characteristics of Nanostructured Materials

Nanostructured materials are attractive in food packaging due to their enhanced functional properties, such as mechanical strength and barrier properties, and a wide range of biologically active compounds, including antibacterial and antioxidant properties, to maintain quality and extend shelf life in various food applications. The advancement of nanomaterials within the food packaging space has brought numerous changes in food preservation, capacity, distribution, and utilization. The characteristics of nanostructured materials are size, shape, specific surface area, aspect ratio, agglomeration/aggregation state, size distribution, solubility, surface morphology/topography, and structure, including crystallinity and defect structure [151].

#### 3.5.2. Analytical Method for Characteristics of Nanomaterials

Nanomaterials can be characterized using a variety of methods, depending on their characteristics. Table 2 below shows different techniques used in analyzing the properties of different nanomaterials. Electron microscopy, electron spectroscopy, field flow fractionation, chromatography, light scattering, Raman spectroscopy, and mass spectrometry have all been used to analyze nanomaterials [152].

#### 3.5.3. Testing of Nanomaterial Food Packaging

Testing food packaging made of nanomaterials at the conceptual stage before being used for food packaging would ensure that materials that fail in design, manufacturing, and customer specifications are rejected to avoid their use. It is necessary to build quality and reliability in the project, phases of production, and distribution. It was found that distributors or suppliers were not very aware of or focused on package evaluation. Different types of nanotechnology packaging materials are quantitatively tested to evaluate their mechanical properties, chemical properties, and geometric properties, while performance tests are evaluated to better simulate the behavior of the packaging material during outdoor transport, storage, and handling.

##### Paper Packaging

Parameters to be tested are square weight, moisture content, thickness, breaking strength, water absorption, breaking length, flexural stiffness, tearing strength, etc. Researchers have produced papers showing the use of nanomaterials in food packaging. Kwon et al., produced sulfate fiber-silver nanoparticle composite sheets with antimicrobial activity against *E. coli*. Muñoz-Shugulí et al., developed β-cyclodextrin complexes containing allyl isothiocyanate. NAMI has announced that it has developed an ecological nano-protective coating that replaces perfluoroalkyl and polyfluoroalkyl substances (PFAS) in paper-based food packaging. PFASs are often mixed with paper pulp or coated on paper to provide water and oil resistance in paper-based food packaging. PFAS is not biodegradable. It is mixed with paper pulp or coated onto paper to provide water and oil resistance to the environment at large. NAMI claims its nano-shield coating is a liquid solution developed through the composition optimization of modified hydrophobic biodegradable polymer and nanoparticles, well dispersed and interacting with each other to form cross-linking to prevent the spread and penetration of water and oil on paper.

##### Plastic Films and Laminates

A lamination film is a layer of a base film such as polyester, nylon, polypropylene, or vinyl bonded with an adhesive. Laminated film is ideal for packing foods such as snacks, coffee, cookies, etc. Laminate film protective barriers are made of PE (LDPE), BOPP, PET, and so on. Plastic films for lamination must be of very good quality. Thickness errors should not exceed ±4% to ±7%. Otherwise, the film will lose its usability in the process and lead to a loss of production. It should be considered that the speed and power of oxygen-removing plastic films and laminated trays are significantly lower compared to iron-based oxygen scavenger bags or tags [128,153]. The tested parameters for plastic films and laminates are thickness, density, tensile strength, impact resistance, duty factor, gloss determination, flexible laminate fog and peel strength determination, elongation at break, etc.

##### Glass Container

The theoretical strength of glass is very high. In practice, the strength is much lower due to surface defects. Nanotechnology has improved the surface of the glass to reduce manufacturing defects and surface coatings due to the use of metal oxides in the glass material, which improves both the electrical and thermal conductivity of the glass [154,155,156]. Three popular classes of glass are standard glass, ceramic glass, and nanostructured glass [157,158]. Closures for glass packaging containers are usually made of metal or plastic and divided into normal seals, vacuum seals, and pressure seals. The parameters tested are color, height, power measurement, mechanical impact force, annealing, thermal shock test, and hydrostatic pressure [159,160].

### 3.6. Types of Plastic Used in Packaging

Carbon nanotubes (CNTs) have recently been synthesized from polymers such as polyvinyl alcohol (PVOH), polypropylene (PP), nylon, polylactic acid (PLA), etc., and have been investigated for packaging purposes, especially as antimicrobial and smart sensors. There are two types of CNTs: single-atom-thick nanotubes and multiple concentric nanotubes. Nanocomposites used in packaging films solve the problems of conventional packaging by providing better antimicrobial, degradation, thermal, barrier, and mechanical properties with a nanosensor that alerts consumers to conditions (e.g., temperature, gas, humidity, impurities, etc.) and food safety [116]. A selection of some of the most important types of plastic used in food packaging is as follows:

#### 3.6.1. Polyethylene Terephthalate (PET or PETE)

PET is widely used in many food packaging applications due to its durability, lightness, and flexibility. Recycling PET bottles saves energy, reduces greenhouse gas emissions, and conserves natural resources. PET containers are used to package foods such as peanut butter, salad dressings, and condiments. Traditional PET production relies on the use of EG and terephthalic acid extracted from petroleum. PET is a thermoplastic polymer, so it can be easily recycled at high temperatures. PET is also easy to recycle, as almost the entire beverage bottle industry uses this plastic. The widespread use of PET in beverage packaging has recently attracted attention due to the short life of this type of container, and particularly its single use. These factors, along with economic and cultural factors, make PET bottles one of the most visible forms of plastic waste [161]. Plastic pollution is now recognized as a global problem [162], and many countries around the world are working to improve local plastic recycling rates [163]. The European Union has decided that by 2030, drink bottles will contain at least 30% recycled plastic. However, PET bottles made from recycled plastic can leach more dangerous chemicals into the new plastic packaging. British researchers report this in a recent study. Researchers from Brunel University in London analyzed 91 studies on food and drinks that contained chemicals from packaging plastics. Of the 193 substances found in PET bottles that end up in food or drink, 150 were found at least once and 18 of them exceeded the legal requirements [164]. Most importantly, and most surprisingly, they found that drink bottles made from recycled plastic appeared to contain more harmful chemicals than pure plastic bottles. The researchers say that recycled PET cannot currently be used as a raw material for food packaging or drink bottles.

#### 3.6.2. Polyvinyl Chloride (PVC)

PVC is widely used as a plastic for food packaging because it is resistant to heat and prevents the growth of microorganisms. It is used to make films, salad bowls, and food trays. PVC requires a variety of hazardous chemicals during production, posing risks to workers, people, and the environment. Evidence shows that PVC is responsible for more national and annual dioxin loads during its lifetime than any other industrial product. Dioxin studies by the United States Environmental Protection Agency (EPA) indicate that there is no safe level of dioxin exposure [165]. Therefore, even a small amount can cause serious health problems [165]. The EPA has also determined that the levels of dioxin currently found in most adults and children are already high enough to pose a health hazard to the American public. The higher the concentration of PVC in the fuel mixture, the higher the formation of dioxins. PVC is important in the formation of dioxins/furans in fires, construction, or land combustion [166]. Nanotechnology strategies are an effective way to improve the dielectric strength, morphology, and surface energy properties of PVC materials. In this study, nanotechnology strategies were used to improve the surface strength properties of polyvinyl chloride (PVC). Different types of nanoparticles, such as clay, ZnO, SiO_2_, and Al_2_O_3_, and concentrations of 1 wt.%, 5 wt.%, and 10 wt.% were investigated [167]. The morphology, dielectric constant, contact angle, wet strength, diffusion coefficient, and adhesion behavior of pure PVC and PVC nanocomposites were investigated. Tap water and salt water were used to study the surface tension properties. The results showed that the type and concentration of nanoparticles used influenced the properties of the nanocomposites obtained. Changes in surface roughness, regulation of hydrophilic expression and dipole/dipole interaction, and changes in the type and concentration of nanoparticles used are the main reasons for improving the surface resistance properties of PVC nanocomposites.

#### 3.6.3. Polystyrene (PS)

Polystyrene could be a lightweight and delicate plastic used for packing materials and disposable food containers. It is additionally utilized within the food industry for items like fast-food packaging. The generation and transfer of PS pose critical natural dangers because it takes a long time to break down and can discharge destructive chemicals into the soil and water. Due to these dangers, numerous companies and organizations are taking steps to stage out the use of PS in their packaging. The consideration of 4% (*w*/*w*) nanoclay brought about an increment in oxygen barrier properties of polystyrene (PS) by 51% in one of the conducted studies [168].

## 4. Shelf Life

### 4.1. Food Preservation and Shelf Life of Current Traditional Materials

Blanching is a commonly used enzyme deactivation process at low temperatures. Enzyme inactivation prevents such reactions from occurring and increases the shelf life. During the heat treatment of fruits and vegetables, the blanching step is similar but aims to block an additional enzyme, so the flotation of fruits or vegetables is reduced [169,170]. The degree of heat treatment necessary to obtain a product with acceptable stability depends on the types of microorganisms and enzymes that are present, the storage conditions of processed foods, and other storage methods used. The production of heat-preserved packaged foods centered on heating the food to reduce it to an acceptable value and retention of its nutrients in airtight packaging to prevent reinfection. Blanching is a process designed to inactivate enzymes and is usually applied immediately before other thermal preservation processes either at high or low temperatures. Without a blanching step, the shelf life of, for example, frozen vegetables used in commercial and domestic practice, but it does slow down storage life, the chemical reactions that cause food spoilage could occur, albeit at a slow rate. In thermal processing of fruits and vegetables, the blanching step is similar, but its objective is to prevent further enzymic breakdown of the foods if delays occur before processing the foods [169,170].

### 4.2. Nanoscale Functionality: How Nanostructured Materials Improve Food Preservation

Food producers are constantly looking for new ways to produce food with improved taste and nutritional properties. Conventional thermal processes reduce the vitamin content of food and affect its structure, taste, and appearance. Nanotechnology provides efficient systems for the reduction or elimination of microorganisms with minimal adverse effects on food ingredients. Nano-encapsulated food ingredients and additives/supplements provide protective barriers, taste and aroma concealment, sustained discharge, and enhanced dispensability for water-insoluble food components and supplements/additives [171]. The use of nano-biocomposites in food packaging has enhanced the ability of food packaging to act as a barrier against gases.

### 4.3. Impact of Nanomaterial Packaging on Food Quality, Shelf Life Extension, and Safety

Nanotechnology has great potential to ensure changes in color, taste, and nutritional values, extend the shelf life of food, and monitor food integrity [172]. Nanotechnology is used in the formation of capsules, emulsions, and biopolymer matrices. Nanoencapsulation hides odors or tastes, regulates the interaction of active ingredients with the food matrix, regulates the release of active ingredients, ensures availability at the target time and a certain speed, and protects them from moisture and heat [173]. Product shelf life is best defined as part of the product development cycle. It is also important that the product packaging requirements are considered in the early stages of product development. Shelf life tests are performed by keeping representative samples of the final product under certain conditions. The process follows those the product encounters from manufacture to consumption. Packaging may limit a product’s shelf life or determine how shelf life-limiting processes are controlled. The shelf life of packaged food is based on acidity, water activity, nutrient content, antimicrobial occurrence, biological structure, temperature, relative humidity, and gas structure [174]. Nano-sized materials are broadly utilized as antimicrobials to decrease the microbial deterioration of packaged foods. The application of nanotechnology also extends to these platforms, including but not limited to the following.

#### 4.3.1. Nano Packaging for Fruits

By mixing polyethylene with nanopowder (nano-Ag, kaolin, anatase TiO_2_, rutile TiO_2_), a new nanopackaging material with lower relative humidity, oxygen permeability, and high longitudinal strength was synthesized, and its effect on the storage quality of the substance was studied. The results showed that nanopackaging was able to maintain the sensory, physicochemical, and physiological quality of strawberries at a higher level than conventional polyethylene packaging bags [175].

#### 4.3.2. Nano Packaging for Beverages

Due to very large aspect ratios, relatively low levels of nanoparticles are sufficient to change the properties of packaging materials without significantly changing the density, transparency, and processing characteristics. The addition of certain nanoparticles to molded objects and films has been shown to make them light, fire-resistant, stronger in terms of mechanical and thermal properties, and less permeable to gases. New packaging solutions focus even more on food safety, preventing the growth of microbes, slowing down oxidation, improving the visibility of violations, and ease of use. Three main categories of nanotechnology applications and functions are being developed for food packaging: improving plastic material barriers; adding active components that can provide functional properties beyond those of conventional active packaging; and identifying and communicating relevant information [176].

#### 4.3.3. Nano Packaging for Chocolates

Nanofilters, which are essentially tiny sieves that can filter viruses and bacteria, are already in use in the brewing and dairy industries. Beet juice was tested in a laboratory where the color was eliminated (turning red wine to white) while maintaining the flavor. With the current technology, lactose can now be filtered out of milk and substituted with another sugar. As a result, food processing may employ less chemicals and heat treatment [177].

#### 4.3.4. Chicken and Spinach Nano Pack

Nanoscale sensors are being developed that monitor toxins and bacteria at all stages of food processing. This helps the producer detect salmonellosis in chickens or *E. coli* in spinach long before the products reach the stores. Self-checkout food packaging is maturing into nanotechnology. When it is connected to a refrigerator, it detects and warns about various chemicals caused by the release of rotten food or the presence of bacteria and then cleans them [152].

### 4.4. Shelf Life Extension: Studies and Findings on Prolonged Freshness

Factors that damage fresh produce and prevent shelf life extension include microbial growth, physical harm, moisture, humidity, temperature, exposure to ethylene, etc. A longer shelf life allows for longer seasonality, less food waste, and a better chance of maintaining freshness. Many studies have been carried out to maintain or extend the shelf life of food products on the market. Some of these studies are highlighted below. For example, Reyes et al., presented interesting results that shed light on the effects of vacuum packaging on the surface color and lipid oxidation of beef fillets. The results of this study suggest that vacuum packaging can be used for beef fillets to maintain quality characteristics for a longer shelf life [178]. A study by Nicosia et al., suggested that there was a tendency to increase or eliminate SSL claims for industrial pesto sauces because the products remained safe for longer than stated on the label. This research will lead to practical results in households to reduce food waste and in the industrial world to reduce inventory turnover and save costs [179]. Panza et al., evaluated the breading of olive paste, a by-product of olive oil production, on fresh fish sticks stored for 15 days at 4 °C. The results showed that enrichment with olive paste increased the total phenolic, flavonoid, and antioxidant activities of the breaded fish samples compared to the control without affecting the sensory parameters [180]. Horticulture professor Avtar Handa found that adding a yeast gene increases the production of a compound that slows the aging and microbial decay of tomatoes. The authors expressed a yeast spermidine synthase (ySpdSyn) gene under constitutive (CaMV35S) and fruit ripening specific (E8) promoters in tomato and determined the alterations in tomato vegetative and fruit physiology in transformed lines compared to the control. The ySpdSyn-transgenic fruits had a longer shelf life, reduced shriveling, and delayed decay symptom development in comparison to the wild-type (WT) fruits. Additionally, the expression of several cell wall and membrane degradation-related genes in ySpdSyn-transgenic fruits was not correlated with the extension of shelf life indicating that the Spd-mediated increase in fruit shelf life is independent of the above factors. Crop maturity, indicated by the percentage of ripening fruits on the vine, was delayed in a CaMV35S-ySpdSyn genotype with fruits accumulating higher levels of the antioxidant lycopene. Together, these results provide evidence for the role of Spd in increasing fruit shelf life, likely by reducing postharvest senescence and decay [181].

### 4.5. Nanocoating Anti-Corrosion Technology for Food Preservation

Nanomaterials within coatings have been shown to provide anti-corrosion properties due to their high hardness, chemical inertness, antimicrobial and anti-fouling properties [182]. Packaged foods, when stored for longer durations in harsh environmental conditions, are susceptible to leaching of acids, alkanes and enzymes that can deteriorate the food coating integrity due to their corrosive nature. This can result in the degradation of food nutrition as well as lead to the toxicity of package contents. Nanobarrier materials provide a solution by impeding the corrosive behavior of food leachants by absorbing them within the matrix or reacting with them to produce benign by-products [183]. Nanomaterials such as ZnO, TiO_2_, Ag, and CuO have shown antibacterial properties to improve stability and the preservation of micronutrients [184]. The incorporation of the above-said materials within food packaging provides a passivation layer to prevent cracking and the leakage of fluids. Nanomaterial-based sensors can alleviate long-term storage deterioration by providing signaling capabilities. These sensors can be tethered with radio frequency identification tags or internet-of-things (IoT)-based interfaces for real-time detection. In addition, the inclusion of sensor-based nanomaterials within the food packaging can detect the spoilage of food items based on the corrosive products of the contents with time-temperature indicators. Single-walled carbon nanotube (SWCNT) composites have been used to detect analytes in dairy and fruit products [185]. An electronic nose can be used to sense volatile compounds based on the aroma of the corrosion products [186]. Thus, a variety of strategies can be employed with nanomaterial-based coatings to detect, mitigate, and neutralize the effects of corrosion products within food packaging.

## 5. Environmental, Safety, and Regulatory Implications

### 5.1. Sustainable Practices: Role of Nanostructured Materials in Green Packaging Solutions

As society leans towards more sustainable practices, nanotechnology has emerged as a promising avenue for making green and sustainable packaging solutions more accessible and efficient. Nanostructured materials, with their unique properties, have demonstrated significant potential in transforming conventional packaging methods, taking us one step closer to a greener planet. Several key nanomaterials have emerged as prominent players in this arena. Montmorillonite (MMT) is utilized to create films that act as robust barriers against oxygen and carbon dioxide. Zinc oxide-coated silicate (ZnO-NPs) serves as a protective shield in sustainable packaging materials, while Kaolinite, a clay mineral, bolsters the packaging’s barrier properties. Silver nanoparticles (Ag-NPs) stand out due to their remarkable antimicrobial properties, thereby effectively prolonging the shelf life of food items. Additionally, titanium dioxide (TiO_2_ NPs) is employed to construct barriers, shielding against various compounds, including oxygen and carbon dioxide. These advancements underscore the pivotal role nanomaterials play in elevating the standards of sustainable food packaging [115]. Polysaccharides like starch, chitosan, and cellulose derivatives are used for their bio-based and antimicrobial properties, as well as their mechanical strength and moisture resistance. Biodegradable polymers such as polylactic acid (PLA), polyhydroxybutyrate (PHB), and polycaprolactone (PCL) offer eco-friendly alternatives to petroleum-based plastics. Edible films made from proteins, lipids, or polysaccharides extend the shelf life of food when consumed with the product [187]. Composite materials, known as nano-enhanced composites, combine these materials’ properties, often incorporating antimicrobial agents to prevent food spoilage. These nanomaterials not only extend food shelf life and enhance safety but also contribute to sustainability by reducing waste from food packaging materials [188].

UV absorption can disturb the flavors, colors, and nutrients in dairy food products, necessitating the need for efficient UV protective solutions [189]. Similarly, crops require protection from direct UV radiation. Properly designed UV protective films can shield not only dairy products but also play a role in preserving chlorophyll synthesis in plants, thus finding application in agriculture [190]. Therefore, another type of biopolymeric nanocomposites under consideration is the incorporation of ultraviolet (UV) protective properties into the packaging materials. By adding biocompatible nanomaterials with maximal absorption properties in the UV spectrum (200–400 nm), the UV protective properties of the packaging materials can be enhanced significantly. These advancements are particularly important in preserving the intrinsic chemical, physical, and biological properties of food items, pharmaceuticals, and beverages during transportation and storage [191].

### 5.2. Biodegradability and Environmental Impact of Nanostructured Materials

The environmental impact and biodegradability of nanomaterials in food packaging are critical considerations for their successful implementation, particularly in disaster-prone areas [192]. While nanotechnology offers innovative solutions for enhancing food safety and quality, the potential environmental consequences of nanomaterials must be carefully evaluated [193]. Organisms are inherently exposed to nanomaterials, yet their mere presence does not guarantee harmlessness. Their impact can be profoundly detrimental, depending on specific circumstances. Additionally, naturally occurring nanoparticles typically aggregate and form larger-sized materials over time, whereas manufactured nanoparticles tend to persist due to the incorporation of surfactants and stabilizers. Consequently, there is a necessity to assess how the utilization of these materials could potentially influence the environment [194]. To categorize a nanomaterial as biodegradable, it is essential to initially identify and chemically characterize it to understand its potential physicochemical properties, which are crucial for evaluating the risks to both human health and the environment. According to Innocenti, the key factors influencing these characteristics encompass composition, structure, molecular weight, vapor pressure, reactivity, solubility in water, boiling and melting points, and stability [195]. The presence of organic compounds in the material’s structure enhances biodegradability, as microorganisms can break down these compounds, leading to material degradation. Additionally, the size and shape of the nanomaterial impact its biodegradability, with smaller particles and irregular shapes being more easily broken down, offering an increased surface area for microbial attack. Researchers can also deliberately modify material structures to impart biodegradability. Ma et al. delved into this concept, concentrating on the application of such modifications to 2D nanomaterials like graphene. Beyond graphene, other 2D materials amenable to modifications include Xenes, Mxenes, transition metal dichalcogenides (TMDs), 2D transition metal oxides (TMOs), and 3D carbon nanotubes (CNTs) [196]. Manatunga et al., have explored chitosan nanoparticles for their antimicrobial properties and potential to extend the shelf life of food products [197]. Chitosan is a biodegradable and biocompatible polymer derived from chitin, with the ability to extend the shelf life of food products and reduce food waste. It offers a sustainable alternative to synthetic polymers in food packaging [197]. Another example is the use of cellulose nanocrystals (CNCs) in food packaging, which have been explored for their potential as a sustainable and biodegradable alternative to synthetic polymers [79]. CNCs are derived from renewable sources such as wood pulp and have been investigated for their mechanical strength, barrier properties, and potential application in active and intelligent food packaging. However, the environmental impact of CNCs and their potential ecotoxicity must also be carefully evaluated to ensure their safe and sustainable use in food packaging.

### 5.3. Reduction of Food Waste: Environmental Benefits

**Figure 3:**
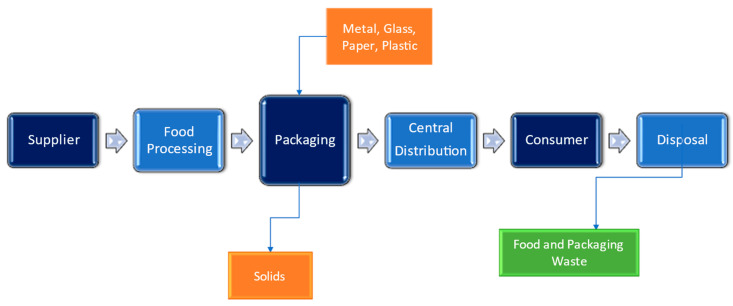
Food production supply chain network diagram.

Reducing food waste through the use of sustainable food packaging materials offers significant environmental benefits. According to the Harvard T.H. Chan School of Public Health, worldwide, one-third of food produced is thrown away uneaten, causing an increased burden on the environment [198]. It is estimated that reducing food waste by 15% could feed more than 25 million Americans every year. The benefits of reducing food waste include cost savings on labor, reduced methane emissions from landfills, and a lower carbon footprint [199]. According to a report by Golden West Packaging, employing environmentally friendly packaging materials, such as recyclable and biodegradable packaging, can reduce landfill waste associated with food packaging. This can lead to a lower carbon footprint and reduced greenhouse gas emissions, contributing to a more sustainable and environmentally friendly food supply chain [200]. By implementing sustainable food packaging materials and reducing food waste, it is possible to mitigate the environmental impact of the food supply chain and contribute to a more sustainable and healthy food system. The food production supply chain network diagram is shown in Figure 3.

### 5.4. Regulatory Framework

#### 5.4.1. Current Guidelines and Regulations for Nanostructured Food Packaging

Several guidelines and regulations have been developed by various regulatory agencies around the world to develop reliable and effective tools to ensure the safe use of nanomaterials. In the context of food, modified nanomaterial is defined in Article 3(2)(f) of the Novel Food Regulation (EU) 2015/2283 as any intentionally produced material that has one or more dimensions of the order of 100 nm or less, or which consists of separate functional parts, either internally or on the surface, having one or more dimensions on the order of 100 nm or less, including structures, agglomerates, or aggregates that may be larger than 100 nm but retain nanoscale properties. In 2021, the European Food Safety Authority [201] published guidance documents on the technical requirements to detect the presence of small particles in food and how to carry out risk assessments of nanomaterials in the food chain, including particle transfer from food contact materials. The office reports on the results of the public consultation. The US Food and Drug Administration reports on advances in nanotechnology and its role in promoting public health through better regulation with a focus on interdisciplinary collaboration and regulatory research. The FDA Nanotechnology Task Force report addresses regulatory and scientific issues and recommends that the FDA consider developing nanotechnology-related guidance for manufacturers and researchers. The FDA has not developed effective guidance on the suitability of current test methods to assess the safety, efficacy, and quality of nanoscale materials in the food industry. The FDA should do more to regulate nanotechnology products and packaging, including the biological effects and interactions of nanoscale materials in the food industry. Developing a nanotechnology guide for manufacturers and researchers would ensure and promote customer confidence in nanotechnology. In 2020, the European Observatory on Nanomaterials (EUON) published a study according to which 87 percent of consumers in five EU countries want better labeling of everyday products containing nanotechnology [202]. In 2019, the European Chemicals Agency published updated versions of two guidance documents for the registration of substances in nanoform.

#### 5.4.2. Comparison of Regulatory Systems for Nanostructured Food Packaging

The regulation of nanomaterials in packaging and other materials varies globally, and the regulatory landscape is continuously evolving. Table 3 below is a comparison of the regulatory approaches in different parts of the world focusing on the United States, the European Union, Canada, Australia, and China.

Overall, while specific regulatory details vary, a common theme across these regions is the focus on safety, health, and environmental concerns, with the industry being held responsible for compliance with relevant standards and regulations.

### 5.5. Safety

#### 5.5.1. Nano-Based Antimicrobial Packaging

These products typically use silver nanoparticles, as well as nano zinc oxide and nano chlorine dioxide. Packaging materials using magnesium oxide, copper oxide, and titanium dioxide in nanoform and carbon nanotubes are also being developed for use in antimicrobial foods [218]. Zinc oxide nanoparticles have been incorporated into several different polymers, including polypropylene [219]. Additionally, zinc oxide effectively absorbs UV radiation without reradiating heat and thus improves the stability of polymer composites. Chitosan is a biopolymer derived from chitin (a polysaccharide component of crustaceans). This has led several groups to investigate its incorporation into various composite materials that could be used in health care and food packaging, including its use as a “green” reagent to reduce and stabilize silver ions [220] in combination with clays such as rectorite used in polymer composites [221,222].

#### 5.5.2. Ensuring Food Safety: Antimicrobial Properties and Pathogen Prevention

Food safety is a public health issue worldwide. The main goal of food safety is to ensure that food does not harm consumers during preparation and consumption [223]. Food must be protected from various physical, chemical, and biological contaminants during processing, handling, and distribution [224]. Advances in nanotechnology have revolutionized the food industry through advances in improving nutritional value, extending shelf life, and reducing packaging waste, including different applications for food processing, safety, and security [224]. Nowadays, due to rapid changes in recipes and eating habits, food safety has become a major concern. Food-borne pathogens, toxins, and other contaminants can have a negative impact on human health. Current methods for detecting pathogens and toxins are labor-intensive and time-consuming. Advances in nanotechnology have rapidly addressed food safety issues of microbial contaminants with improved toxin detection, shelf life, and packaging strategies [60]. In addition, nanomaterials, including metal nanoparticles, carbon nanotubes, quantum dots, and other active nanomaterials, can be used to develop bioassays for microbial measurements and other tests for food safety applications [60,61]. Food labeling is currently required to reduce the risk of consumers ingesting the contents of oxygen-free bags or other active ingredients in the package. Some active packages may differ from inactive packages. Therefore, it may be advisable to use appropriate notation to explain this to the consumer, even if there are no regulations. The use of natural antimicrobial foods can ensure food safety and quality as opposed to other preservation systems such as chemical or thermal preservation systems. The demand for natural antimicrobial agents to replace synthetic ones is expected to increase [56]. Antimicrobial agents are slowly released into food or the atmosphere over the food and prevent microbial growth during its short shelf life [57]. One of the investigated applications is the use of carbon nanotubes (CNTs) as antimicrobial agents for water disinfection. CNTs have been widely studied as promising antimicrobial agents due to their stability and effective biological properties [58,59]. The use of ethanol as an antibacterial agent is effective against mold and also inhibits the growth of yeast and bacteria. Many factors influence the antimicrobial effect of carbon dioxide, especially microbial load, gas concentration, temperature, and permeability of the packaging film. Packaging materials designed to be antimicrobial inhibit microbial growth but rarely act alone as the major shelf-limiting factor. Antimicrobial action can be achieved in two ways. The release of conservatives or portable systems contains a preservative that is designed to be portable food [225]. Several antimicrobial agents are commercially available, and their activity and efficacy have been reviewed [220]. An example of this technology is Microban from Microban Products Co., Melton Mowbray, UK with two locations in Staffordshire and Leicestershire. This product incorporates the biocide triclosan into almost all types of plastic so that it moves freely to the surface to kill any bacteria that may appear. Table 4 lists some commercial antimicrobial agents.

**Figure 4:**
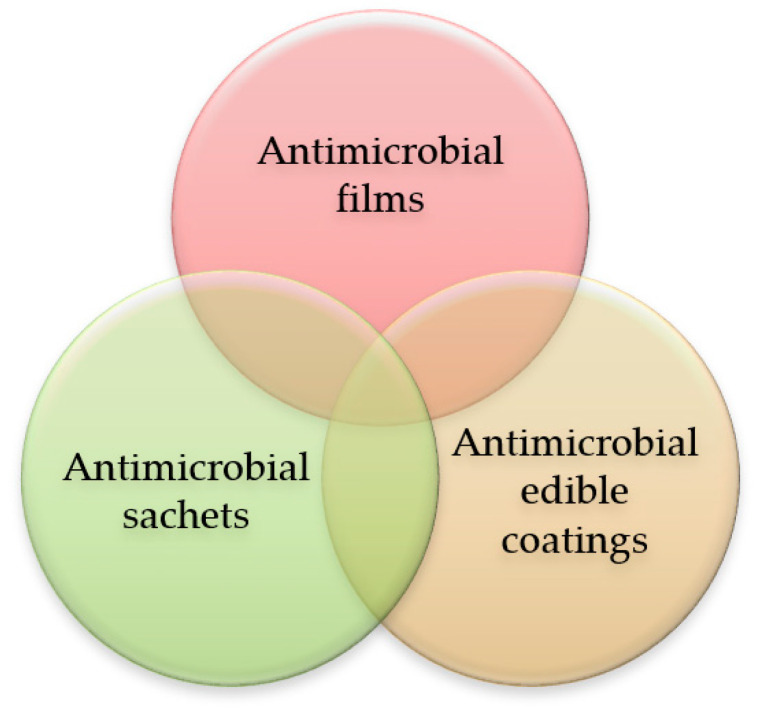
Types of antimicrobial agents.

Before the production of antimicrobial agents, the surface of plastic films is often modified to improve the adhesion of the antimicrobial agents to the polymer matrix [97]. The design of an antimicrobial coating requires detailed information about interactions between the active ingredient, the coating, the substrate, and the food. Specifically, the active coating must adhere effectively to the film base and be inert in direct contact with food and the concentration of the released active ingredient must be controlled. Three types of antimicrobial agents were documented for use in fresh and minimally processed fruits and vegetables [89]. The antimicrobial agents are shown in Figure 4.

Packaging materials designed to have antimicrobial activity provide a hurdle for microbial growth but seldom act alone as the key shelf life-limiting factor. Antimicrobial activity can be obtained in two ways. Preservative-releasing or migrating systems contain a preservative intended for migration into the food [225].

#### 5.5.3. Effects of Nanomaterials on the Human Body

A variety of acute and long-term consequences, inflammation, and carcinogenesis are linked to nanoparticle exposure [226]. Human cells may experience oxidative stress, liver and kidney damage, and DNA damage because of prolonged nanoparticle exposure [227]. Numerous studies have shown that injected, infused or inhaled nanoparticles can travel to various organs and tissues after entering the systemic circulation [228]. It is very common for people working in nanotechnology plants to inhale particles and for those particles to penetrate their skin [229]. The occurrence of toxic effects on the exposed human body and the phenomenon of migration is closely associated with the toxicological risk associated with the use of nanomaterials in food packaging [230]. Metals usually migrate when food encounters materials used for packaging. The migrant nanoparticle diffuses, dissolves, and disperses throughout the food as part of the migration process. For example, a researcher found that the migration of nanoparticles, such as AgNPs from baby products, can be detrimental to the health of infants [231]. Determining the potential health effects of nanoparticles when they come into contact with food products requires an understanding of how they migrate [232]. International regulations have not yet been able to be adopted due to limited information on the potential toxicity of nanomaterials [233].

#### 5.5.4. Safety Assessment Protocols: Evaluating Nanomaterials for Food Contact

The European Food Safety Authority [201] has released guidance documents on the technical requirements for the measurement and risk assessment of nanomaterials in the food and feed chain [207]. The guidance covers physicochemical characterization, exposure assessment, and hazard identification of nanomaterials. It includes a tiered framework for toxicological testing, addressing aspects like genotoxicity, local and systemic toxicity, and potential effects on the gut microbiome and endocrine activity. Additionally, the guidance discusses the use of read-across and integrated testing strategies to fill data gaps and inform risk characterization and uncertainty analysis [201].

#### 5.5.5. Ethical Considerations: Balancing Innovation with Safety in Humanitarian Food Packaging

There are currently no universally accepted standards on the biodegradability of nanomaterials, as the biodegradability of a material can depend on a variety of factors such as the specific type of material, its chemical functionalization, and the conditions in which it is disposed of [234]. However, organizations such as the American Society for Testing and Materials (ASTM) and the International Organization for Standardization (ISO) have developed guidelines and protocols for testing the biodegradability of materials in various conditions [235]. These guidelines typically involve assessing the material’s ability to degrade under specific environmental conditions, such as in soil or water, and measuring the rate and extent of degradation. Additionally, some organizations have developed standards for biodegradable plastics, which may be used as a reference for the biodegradability of nanomaterials. Still, it is important to keep in mind that they are not specific to nanomaterials and might not always be applicable [235]. The ISO TC 229 has published a Technical Report outlining best practices for occupational health and safety regarding nanomaterials and nanotechnologies. This report includes recommendations for toxicology testing, risk management, exposure control, and safety data sheets [192]. A collaborative effort between researchers, technologists, practitioners, and regulators is necessary to ensure the safe development of nanoproducts. Different approaches, such as those established by public standards-setting bodies and private enterprises, can be considered to manage the risks associated with using nanomaterials [235].

## 6. Nanoengineering and Humanitarian Considerations

### 6.1. Nanoengineering and Food Security Challenges in Vulnerable Regions

Food security challenges in food-insecure and vulnerable populations are complex and multifaceted, requiring comprehensive and sustainable solutions. Vulnerable populations, such as those in remote and underserved communities, often face significant obstacles in accessing safe and nutritious food. The food environment in these regions is characterized by the limited availability of fresh produce, inadequate sanitation, and poor education and training on food safety, leading to heightened food safety risks [49]. Additionally, vulnerable populations are more susceptible to becoming food insecure or worsening their food security in the face of shocks, such as crop failures, loss of income, or sudden health crises, and have fewer coping strategies to deal with these shocks. Achieving food security in vulnerable populations, particularly in the context of extreme events such as droughts or disasters, is a critical challenge that requires sustainable and effective interventions. Pastoralist communities, for example, are among the most vulnerable to hunger when faced with extreme events, highlighting the need for targeted and context-specific interventions to improve food security in these populations [236]. The relationship between health and food security is also complex, as medical services play a crucial role in treating severe malnutrition and preventing illnesses from becoming prolonged, which can affect the ability to work and further compromise food security and health [236]. Research priorities for global food security under extreme events have been identified as a key area for future research, providing a prioritization of threats to global food security from extreme events and emerging research questions that require further investigation [45]. Addressing the complex and interconnected challenges of food security in vulnerable populations requires a comprehensive and multidisciplinary approach, encompassing areas such as food environment policymaking, food safety, value chain approaches, and sustainable solutions to improve access to safe and nutritious food in these regions. Food viability is an important factor to consider when transporting food to remote and underserved communities. Fresh produce, such as vegetables and fruits, is more nutritious than canned food and is essential for addressing malnutrition and improving the health of vulnerable populations. However, the transportation of fresh produce to remote areas presents logistical challenges, including the need for proper storage and transportation facilities to maintain the freshness and quality of the food aid [237].

### 6.2. Addressing Food Security: The Role of Nanoengineering in Underprivileged Regions

Nanotechnology is a promising solution to tackle food security challenges in vulnerable regions, particularly when it comes to transporting nutritious food to remote and underserved communities. The application of nanomaterials in food processing, preservation, and packaging stands is a key enabler for ensuring the secure and fresh delivery of food aid to these regions. The application of nanomaterials in the food sector is recognized as a pivotal area for future innovations, holding the potential to enhance the bioavailability and retention of active biochemicals in food. This improvement results in increased loading capacity and heightened stability [238]. Moreover, the adoption of nanotechnology in agriculture and the food industry is acknowledged as a crucial strategy to safeguard food security and safety, especially in the face of global climate change and rapid population growth [239]. Furthermore, the incorporation of nanotechnology in agriculture offers a potent approach to addressing prevailing food security challenges and threats, especially within the context of sustainable agriculture and crop enhancement [240]. The ongoing global food crisis, exacerbated by factors such as climate shocks, regional conflicts, and the pandemic, underscores the pressing need for swift policy actions to mitigate the impact of elevated import costs for food and fertilizer on food insecurity. In this critical context, the application of nanotechnology in ensuring food security plays a vital role in guaranteeing the safe and fresh delivery of food aid to vulnerable regions. This contribution becomes integral to alleviating human suffering and safeguarding the most vulnerable populations.

### 6.3. Disaster-Prone Areas: Emergency Food Supplies and Nanoengineered Packaging

Nanotechnology offers innovative solutions for delivering food aid over long distances in disaster-prone areas and emergency situations. Nanomaterials can be employed in food packaging to extend the shelf life of food items, maintain food freshness, and preserve the taste and quality of the food during transportation and storage [241]. According to Durán and Marcato, nanoengineered packaging materials provide improved mechanical barriers, heat resistance, and biodegradability than traditional food packaging, making them well-suited for preserving food in disaster-ridden areas [242]. The application of nanotechnology in food packaging is classified based on its function, with most nanoparticles used in food packaging possessing antimicrobial capabilities and acting as antimicrobial polypeptide carriers to protect against microbial deterioration. Nanoparticles can be incorporated into packaging materials to provide protection, tamper resistance, and specific physical, chemical, and biological properties, ensuring the safety and quality of the packaged food [243]. Additionally, nanosensors can be employed to identify infections or contamination in food throughout manufacturing, processing, packaging, storage, and transport, enhancing food safety and quality assessment [244]. Nanotechnology-derived food packaging materials have the potential to address the challenges of delivering food aid to disaster-prone areas and vulnerable communities. These materials can help maintain the freshness and quality of food items during transportation and storage, ensuring the availability of safe and nutritious food in emergencies [129]. The use of nanomaterials in food packaging represents a significant advancement in food security and safety, offering sustainable solutions to preserve food and mitigate the impact of disasters on vulnerable populations [245].

### 6.4. Case Studies: Successful Implementation in Challenging Environments

Several instances and case studies demonstrate the successful implementation of nanomaterials in food packaging, food safety, and food processing, particularly in challenging environments such as disaster-prone areas. Nanotechnology has been applied to develop improved packaging, active packaging, and intelligent packaging, offering enhanced mechanical barriers, antimicrobial properties, and improved shelf life for food products [79]. In a study published in *Frontiers in Microbiology*, the application of nanotechnology in the food industry was highlighted, emphasizing the significant difference nanomaterials bring to food quality, safety, and health benefits. Nanomaterials have been utilized in food processing, packaging, and sensing, providing improved mechanical strength, barrier properties, and antimicrobial films, as well as nanosensing for pathogen detection and ensuring food safety [129]. Furthermore, research published in the journal *Frontiers in Microbiology* discussed the use of nanotechnology in food packaging, preservation, and safety assessment. The study highlighted the role of nanotechnology in ensuring food safety by preventing decomposition and loss of nutrients, resulting in a longer shelf life for food products [243].

### 6.5. Implications for the Future: Prospects of Nanoengineering in Humanitarian Food Packaging

The future of nanotechnology in food packaging holds great promise for enhancing food safety, quality, and shelf life, particularly in challenging environments such as disaster-prone areas [187]. Nanotechnology is a rapidly advancing field that offers opportunities for the development of new nanomaterials and nanosensors, which can significantly impact the food industry, including food packaging, food security, and food processing [55]. The application of nanotechnology in food packaging has the potential to provide improved mechanical barriers, detection of microbial contamination, and potentially enhanced bioavailability of nutrients, offering innovative solutions for delivering food aid to disaster-prone areas and vulnerable communities [79]. The relevance of nanomaterials in food packaging and its advanced prospects has been highlighted in a study published in the *Journal of Nanotechnology*, emphasizing the significant difference nanomaterials bring to food quality, safety, and health benefits. Nanomaterials have been utilized in food processing, packaging, and sensing, providing improved mechanical strength, barrier properties, and antimicrobial films, as well as nanosensing for pathogen detection and ensuring food safety [181]. Additionally, the application of nanotechnology in food packaging has been explored for the controlled release of preservatives and antimicrobials, extending the product shelf life within the package, and ensuring the real-time quality of food products. Nanotechnology-based food packaging materials have been instrumental in addressing food quality, safety, and stability concerns, offering numerous advantages over conventional food packaging [55].

## 7. Conclusions

This paper explores the design, manufacturing, regulatory, and sustainability implications of engineered nanomaterial coatings for food packaging. The importance of food packaging and the challenges associated with traditional packaging materials are outlined. The potential benefits of nanomaterial coatings, including improved mechanical and barrier properties, as well as the ability to incorporate active ingredients for antimicrobial and antioxidant effects are examined. The regulatory landscape for nanomaterials in food packaging, including the current lack of universally accepted standards for the biodegradability of nanomaterials are discussed. Further, the efforts of organizations such as the American Society for Testing and Materials (ASTM) and the International Organization for Standardization (ISO) to develop guidelines and protocols for testing the biodegradability of materials in various conditions are highlighted. The importance of toxicology testing, risk management, exposure control, and safety data sheets in ensuring the safe development of nanoproducts is delineated. Thus, there is a need for a collaborative effort between researchers, technologists, practitioners, and regulators to manage the risks associated with using nanomaterials. In conclusion, engineered nanomaterial coatings have the potential to address many of the challenges associated with traditional food packaging materials. However, the safe and sustainable use of nanomaterials in food packaging requires the careful assessment and consideration of various factors, including biodegradability, safety, and regulatory compliance. At present, minimal standards exist to govern the design, development, and manufacture of nanomaterial coatings. Moving forward, it will be important to continue to develop and refine the guidelines and protocols for the use of nanomaterials in food packaging to ensure the safety and sustainability of our food supply. Finally, targeted R&D investments can aid in the translation of nanomaterials and nanosurface coatings with increased levels of automation.

## Figures and Tables

**Table 1:** Nanomaterials used in food contact materials.

Nanomaterial	Property Enhancement	Applications
Titanium nitride	Improvement of thermal properties [119], antimicrobial and deodorant agent [120], UV filter [121]	PET, fridges [120]
Zinc oxide	UV filter, antimicrobial and fungi static agent [122], deodorant [120]	Plastic glasses, plastic films
Carbon black	Additive [123]	Rubber, silicones, printing inks
Silver	Antimicrobial [124], anti-biotic, antistatic agent	Reusable food containers [120]
Nanoclay (bentonite)	Improvement of barrier properties [125]	PE, PET, PP, PS, TPO and nylon [120,126]
Aluminum	Filler in polymers, scratch- and abrasion-resistance in coatings [123], improvement of barrier properties, UV filter [120]	
Silicon dioxide	Anti-slip agent	Printing inks, paper and boards, rubbers, silicones

**Table 2:** Properties of nanoparticles (nanoparticle powder).

Size, size distribution, shape [152]	Scanning electron microscopy (SEM)
	Transmission electron microscopy (TEM)
	Atomic force microscopy (AFM)
	X-ray diffraction (XRD) for crystalline nanoparticles
	Differential mobility analysis
Crystallinity, crystal structure [152]	X-ray diffraction (XRD)
	Electron diffraction in a transmission electron microscope (ED)
Chemical composition and purity of a nanoparticle ensemble (powder sample)	Inductively coupled mass spectroscopy (ICPMS)
	Inductively coupled plasma atomic emission spectroscopy (ICP-AES)
Chemical properties of single nanoparticles [152]	Atom-absorption spectroscopy (AAS)
	X-ray fluorescence spectroscopy (XRF)
	X-ray photoemission spectroscopy (XPS)
	Time-of-flight secondary ion mass spectroscopy (TOF-SIMS)
	Ultraviolet-visible spectroscopy (UV-Vis)
	Fourier-transform infrared spectroscopy (FTIR)
	Energy-dispersive (wavelength) dispersive X-ray spectroscopy in an electron microscope
Surface chemistry and surface reactivity [152]	X-ray photoemission spectroscopy (XPS)
	Electron spin resonance (ESR)
	Auger electron spectroscopy (AES)
Surface area as an indicator for agglomeration [152]	Isothermal gas adsorption/BET

**Table 3:** Regulatory systems for nanostructured food packaging in the world.

Criteria	United States	European Union	Canada	Australia	China
Regulatory Body	FDA (Food and Drug Administration), EPA (Environmental Protection Agency) [203]	European Chemicals Agency (ECHA), European Food Safety Authority (EFSA) [204]	Health Canada, Environment nada [205]	National Industrial Chemicals Notification and Assessment Scheme (NICNAS), Food Standards Australia New Zealand (FSANZ), Therapeutic Goods Administration (TGA), National Industrial Chemicals Notification and Assessment Scheme (NICNAS) The Australian Competition and Consumer Commission (ACCC) [206]	State Food and Drug Administration (SFDA) The National Health Commission of China, The National Medical Products Administration (NMPA) (Yu) National Nanotechnology Standardization Technical Committee (NSTC) Standardization Administration of China/Technical Committee (SAC/279) [207,208]
Responsibility	The FDA is responsible for ensuring products meet safety standards and comply with legal requirements. [203,209] EPA regulates nanomaterials under TSCA, requiring reporting and recordkeeping for nanoscale chemical substances [210,211]	Nanomaterials are assessed to ensure that their uses are properly evaluated, and any risks are controlled. [212] EFSA provides guidance on risk assessment which includes considerations for nanomaterials in food and Food Contact Materials (FCMs) [207]	Health Canada does not regulate nanomaterials in these products [131,203].	FSANZ regulates nanotechnologies in food. TGA manages nanoparticles in therapeutic goods and medical devices [213]. NICNAS is responsible for industrial nanomaterials in consumer goods and coatings The ACCC regulates all consumer products containing nanomaterials that do not fall under other regulatory jurisdictions [214].	The SAC/TC279 serves as the coordinating body to draft essential nanotechnology standards. [208] These bodies are responsible for managing risks and appropriate frameworks for nanotechnology
Legislation	Federal Food, Drug, and Cosmetic Act, Toxic Substances Control Act (TSCA)	REACH, CLP, Biocidal Products Regulation (BPR) [119]	Framework for risk assessment of manufactured nanomaterials under CEPA 1999 [205]	Food Standards Australia New Zealand (FSANZ)—under the joint Australia New Zealand Food Standards Code (the Code) [213]	Environmental Administration of New Chemical Substances made effective in September 2003 [208] Standard for the Use of Food Additives (GB 2760-2014), General Standard for the Labeling of Prepackaged Foods (GB7718-2011), Standard for the Nutritional Labeling of Prepackaged Foods (GB 28050-2011). [215]
Labelling Requirements	FDA recommends voluntary labeling for cosmetics [216]	Mandatory labeling for certain nanomaterials in cosmetics and other products [204]	Labeling not explicitly required for nanomaterials [131,205]	The Code mandates that food packaging must be safe and reported [206]	Mandatory labeling and reporting requirements [208,217]
Product Bans or Restrictions	Varies by product type; e.g., certain sunscreens with nanomaterials regulated [211,216]	Bans or restrictions on certain nanomaterials in cosmetics [119]	General guidance and risk assessment for products containing nanomaterials are under existing legislative frameworks [205]	Some restrictions on nanomaterials transferring from packaging to food [213]	Stringent regulations on the use of nanomaterials in cosmetics. - Requirement for specific physicochemical indicators and toxicological test data for nanomaterials. - Use of nanotechnology in children’s cosmetics is restricted [217]
Reporting of Nanomaterial Use	EPA requires reporting [211]	Mandatory reporting under REACH [207]	Reporting under CEPA [205]	Reporting required [206]	Mandatory reporting [208,215]

**Table 4:** List of some commercially antimicrobial agents.

Company	Product Name	Active Ingredient
Bactiblock, Zaragoza, Spain	BactiBlock 920 B4	silver ions-based
Nanopack Technology & packaging, Girona, Spain	Aglon	Silver zeolites
BASF, Beaumont, TX, USA	Irgguard	Silver
Tessara-Grapetek, Cape Town, South Africa	Uvacy ^TM^	Sulphur dioxide

## Data Availability

The data presented in this study are available on request from the correspondence author.
